# Circulating tsRNAs serve as potential biomarkers for predicting postoperative delirium in elderly patients receiving lower extremity orthopedic surgery

**DOI:** 10.3389/fpsyt.2025.1522984

**Published:** 2025-03-26

**Authors:** Angyang Cao, Rui Zhao, Chunqu Chen, Can Wu, Yiwei Zhang, Changshun Huang, Binbin Zhu

**Affiliations:** ^1^ Department of Anesthesiology, The First Affliated Hospital of Ningbo University, Ningbo, China; ^2^ School of Medicine, Ningbo University, Ningbo, China; ^3^ Department of imaging, Hubei Provincial Hospital of Traditional Chinese Medicine, Wuhan, China; ^4^ Department of Clinical laboratory, Jinhua Maternal and Child Health Care Hospital, Jinhua, China

**Keywords:** tsRNA, postoperative delirium (POD), RT-qPCR, elderly patients, propensity score matching (PSM)

## Abstract

**Background:**

Postoperative delirium (POD) is a serious neuropsychiatric complication in elderly surgical patients, yet its pathogenesis remains incompletely understood. Transfer RNA-derived small RNAs (tsRNAs) have emerged as crucial regulators in neurological disorders. We investigated whether specific tsRNAs could serve as predictive biomarkers for POD.

**Methods:**

This study conducted a prospective case-control study of 158 elderly patients (≥60 years) undergoing orthopedic surgery. Plasma samples were collected preoperatively and on postoperative day 3.tsRNA expression profiles were analyzed using RNA sequencing and validated by RT-qPCR. Propensity score matching was performed to balance demographic and clinical variables. The predictive value of candidate tsRNAs was assessed using ROC analysis, and their potential functions were explored through bioinformatic analyses.

**Results:**

Among 128 non-POD and 30 POD patients, two tsRNAs (Other-14: 31-tRNA-Gly-CCC-3 and Other-39: 73-tRNA-Arg-TCG-5) showed significantly elevated preoperative levels in POD patients (p<0.001).ROC analysis revealed strong predictive performance (AUC=0.868 and 0.956, respectively).These differences persisted in the propensity-matched cohort (29 pairs).Bioinformatic analyses indicated enrichment in pathways related to neurotransmission, inflammation, and metabolism.

**Conclusion:**

This study identified novel tsRNA biomarkers that robustly predict POD risk and provide insights into its molecular pathogenesis. These findings may facilitate early risk stratification and preventive interventions.

## 1 Introduction

Postoperative delirium (POD) is a common and serious condition characterized by acute changes in consciousness, cognition, and behavior in elderly patients after surgery (1, 2). The symptoms manifest acutely, typically emerging 1-3 days after the surgery (3). POD has deleterious effects on both early and long-term prognosis. Studies have revealed that individuals who experience delirium are at a heightened risk for complications following a surgical procedure, as well as perioperative mortality and prolonged hospitalization, which consequently leads to escalated medical expenses during their hospital stay (4). Long-term follow-up studies have indicated that delirium patients have an elevated incidence of postoperative cognitive dysfunction, lower quality of life and increased long-term mortality (5). Currently, the diagnosis of POD relies on neuropsychological scales, which are time-consuming, subjective, and prone to inaccuracies, such as missed or misdiagnosed cases. There is an urgent need for reliable and easily applicable biomarkers to improve early detection and intervention. Therefore, early detection, diagnosis and intervention of POD are of great significance to improve the long-term prognosis and quality of life of postoperative patients.

Despite POD being a prevalent and severe complication among elderly patients after surgery, the precise pathogenesis of POD remains elusive. Potential contributing factors include central nervous system inflammation (6), oxidative stress (5, 7), central cholinergic system disruptions (8), and sleep disturbances (9). Notably, studies have shown that preoperative cognitive decline, particularly in the context of the aging brain, serves as an independent risk factor for POD development (10), and the “fragile brain” caused by neuronal aging is at increased risk of POD in the face of surgical stress. Alzheimer’s disease (AD) is the most common and widely studied neurological disorder affecting the central nervous system. Aβ and tau are the characteristic markers of AD. Studies have demonstrated that alterations in the levels of Aβ and tau in cerebrospinal fluid prior to surgery can predict the occurrence of POD to a certain extent. The correlation between POD and Alzheimer’s disease (AD) markers, such as cerebrospinal fluid Aβ and tau levels, suggests shared pathological pathways (11, 12).

tRNA (or transfer RNA) is a well-defined non-coding RNA that mediates translation and regulates non-translational cellular processes. Vascular endothelial growth factor (ANG) and Dicer, among other specific nucleic acid endonucleases, cleave precursor or mature tRNA under specific stress conditions, resulting in various subtypes of tsRNA, primarily tRNA-derived RNA fragments (tRF) and tRNA half-molecules (tiRNA) (13). Based on the existing research, tRFs and tiRNAs are implicated in crucial life activities such as cell proliferation, the initiation of viral reverse transcriptase, the regulation of gene expression, RNA processing, the modulation of DNA damage responses (14–17). Previous research has demonstrated that the aberrant expression of tsRNA is strongly correlated with neurodevelopmental disorders and neurodegenerative diseases (13, 18, 19), and it is capable of participating in the genesis and progression of AD by modulating the stability of mRNA, transcription, and protein translation processes (13, 20). Zhang et al. reported that tsRNAs influence neurodevelopmental and neurodegenerative processes, particularly in AD through regulation of Aβ peptide formation and astrocyte network function (21). High-throughput analyses have revealed that altered tsRNA expression, such as reduced 5’ tsRNA-Tyr levels, may sensitize neurons to oxidative stress (22). Liu et al. identified significant glutamine tRNA fragment accumulation in aged brains’ mitochondria, impairing mitochondrial protein translation and inner membrane integrity, which disrupts glutamate synthesis and accelerates brain aging and memory deterioration (23). As a category of stable circulating biomarkers, certain tsRNAs modulate gene expression through influencing key pathways implicated in neural plasticity and neuroinflammatory responses (18). However, despite the fact that tsRNAs have shown prominent prospects as biomarkers and therapeutic targets in other neurological disorders (24), their potential function in POD remains uninvestigated.

While cerebrospinal fluid most directly reflects central nervous system changes, its collection is invasive and clinically impractical. Peripheral blood offers a more accessible alternative, particularly as POD-associated inflammation and oxidative stress can compromise the blood-brain barrier, allowing central nervous system markers to enter circulation (25). Recent studies indicate that non-coding RNAs function as intercellular messengers in various biological processes beyond protein synthesis. In stress scenarios like trauma, ischemia, and hypoxia, injured cells may release extracellular RNA (26). Additionally, Non-coding RNA can be transported to remote cells through extracellular microvesicles (27).Therefore, as a relatively accessible fluid sample of the body, peripheral blood carries biological information that reflects the pathological alterations of central nervous system injury, which is conducive to the study of POD-related mechanisms. Importantly, tsRNAs demonstrate high stability and abundance in plasma compared to other non-coding RNAs such as lncRNAs and miRNAs (28, 29), making them promising candidates as circulating biomarkers.

This study aims to investigate the potential of circulating tsRNAs as predictive biomarkers for POD in elderly patients undergoing lower extremity orthopedic surgery. Using microarray technology, we analyzed plasma tsRNA expression profiles to identify POD-associated signatures that could elucidate pathophysiological mechanisms and facilitate early risk assessment. This research may provide new insights for improving postoperative outcomes in high-risk elderly patients through better prediction and prevention strategies.

## 2 Materials and methods

### 2.1 Patients and blood sample collection

**Figure 1:**
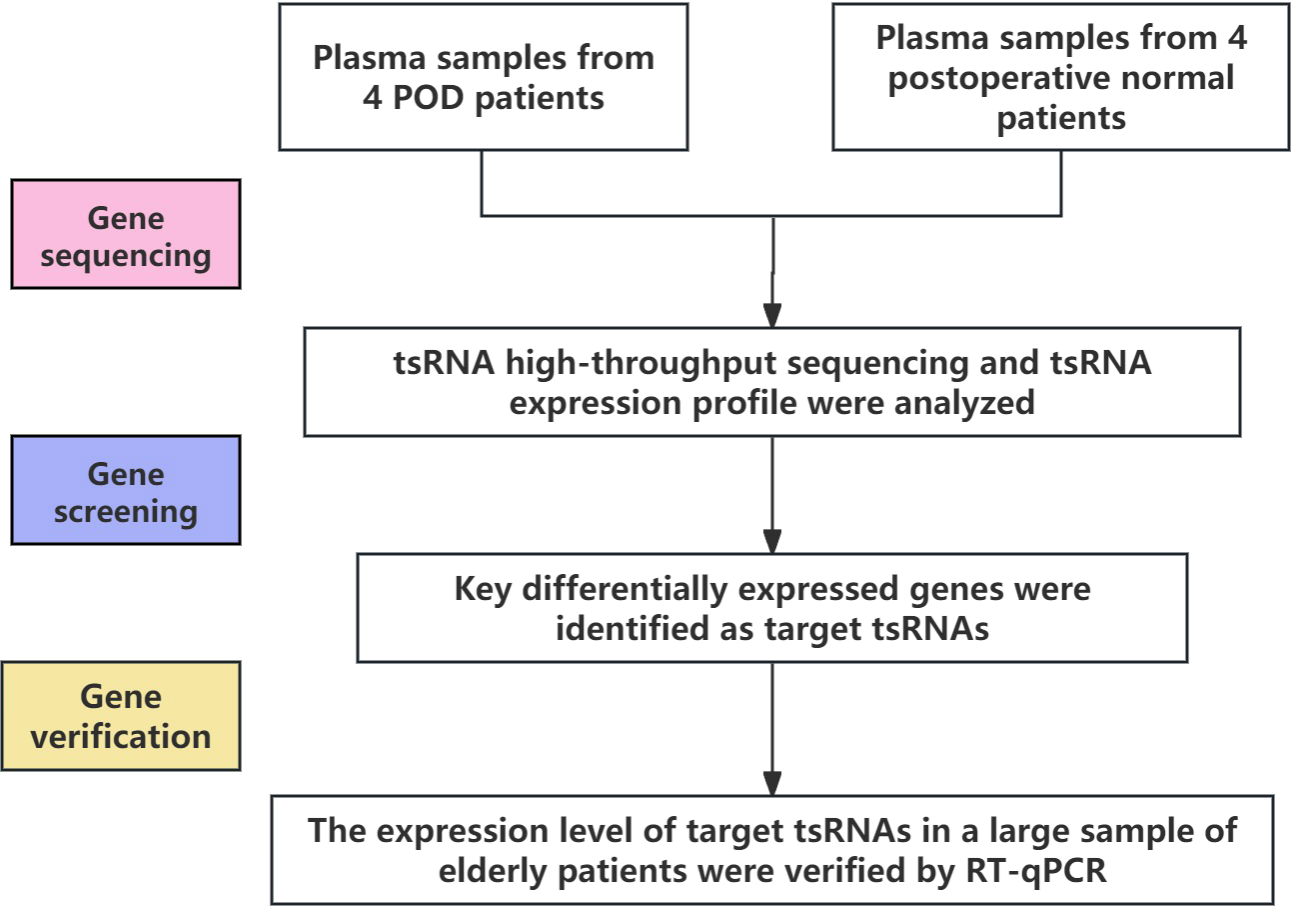
The technical roadmap of this study.

This case-control study included elderly patients aged 60 years and older who underwent orthopedic surgery at the First Affiliated Hospital of Ningbo University from August 2022 to December 2023. Patients diagnosed with POD by unconsciousness assessment within 3 days after surgery were classified as POD group. Peripheral blood of all subjects was collected before surgery with the approval of ethics Committee and written informed consent. In this study, Shanghai Kangcheng Company was commissioned to perform RNA sequencing on plasma samples from 4 patients with postoperative delirium and 4 normal patients to screen out the differentially expressed ts RNA related to postoperative delirium. However, tsRNA-14:31-tRNA-Gly-CCC-3 and tsRNA-39:73-tRNA-Arg-TCG-5 were confirmed as the research objects of this study. Then real-time fluorescent quantitative PCR (RT-qPCR) was used to verify the differentially expressed tsRNAs identified by sequencing in plasma samples from a large population (**Figure 1**).

Inclusion criteria: Patients having elective orthopedic surgery at our hospital; age ≥60 years; ASA grade I-III; no history of substance abuse. Exclusion criteria: total score of daily living scale ≤23 points; other neurological diseases (such as stroke, Parkinson’s disease, Alzheimer’s disease, etc.) and cognitive decline caused by vascular or trauma; patients with severe respiratory, circulatory or other system dysfunction; patients with low compliance, severe hearing and visual impairment, and reading and comprehension impairment unable to cooperate with CAM scale assessment.

### 2.2 Anesthesia protocol

The selection of anesthesia depends on the patient’s desires, circumstances, and the skills of the anesthesiologist and surgeon. Two options are tracheal intubation general anesthesia or subarachnoid block. Vital signs were promptly monitored upon the patient’s arrival in the operating room, and oxygen therapy was given at a rate of 5 L/min. General anesthesia is induced using specific drugs, including midazolam, propofol, etomidate and sufentanil. After induction and intubation, the anesthesia is maintained with remifentanil, propofol, and sevoflurane. Vasoactive medications are adjusted based on the patient’s vital signs. Subarachnoid spinal anesthesia is administered as a single dose without an epidural catheter.

### 2.3 Diagnosis of postoperative delirium

Delirium was assessed using the Confusion Assessment Method (CAM) scale twice daily (morning and afternoon) for the first 3 postoperative days. The presence, severity, and duration of delirium were recorded. Additional delirium assessments included a 24-hour history review and a home version of the CAM administered to family members. Patients whose delirium resolved within 3 days after surgery completed the follow-up assessment. However, patients with persistent delirium beyond postoperative day 3 were continuously monitored until the delirium resolved or they were discharged from the hospital.

### 2.4 Data collection

Demographic data collected included age, education, weight, height, BMI. Intraoperative data included anesthesia type, surgical duration, blood loss. Preoperative and postoperative day 3 cognitive scores and pain (visual analog scale) were measured. Peripheral venous blood samples were collected in EDTA tubes one day before and after surgery following overnight fasting. Samples were kept at room temperature for 1-2 hours then centrifuged at 3000 rpm for 15 minutes at 4°C. The upper plasma layer was carefully pipetted off and transferred to RNase-free tubes for storage at -80°C until analysis.

### 2.5 RNA extraction and quantitative RT-PCR analysis

Total RNA was extracted from 500 μL plasma samples using TRIzol LS reagent (Invitrogen, USA) according to the manufacturer’s protocol. Briefly, plasma was homogenized with 1 mL TRIzol LS, treated with 300 μL chloroform, centrifuged at 12,000 x g for 15 min at 4°C to separate the aqueous phase, and RNA was precipitated using 650 μL isopropanol. The RNA pellet was washed with 75% ethanol, air-dried, and resuspended in 10-20 μL RNase-free water. RNA concentration and purity were analyzed using a NanoDrop 2000 spectrophotometer (Thermo Scientific, USA). An OD260/280 ratio above 1.8 was considered acceptable. The cDNA synthesis was performed using MMLV reverse transcriptase (Shanghai GenePharma Co., Ltd) and RT-qPCR was carried out using SYBR Green PCR Master Mix (Shanghai GenePharma Co., Ltd). The cycling conditions were: 95°C for 3 min;40 cycles of 95°C for 12 s, 62°C for 40 s; and melt curve analysis. U6 snRNA was used as an internal control. The relative levels of tsRNA-14:31-tRNA-Gly-CCC-3 and tsRNA-39:73-tRNA-Arg-TCG-5 were analyzed by the 2−ΔΔCt method. RT -qPCR primers are listed in **Table 1**.

**Table 1:** The primers and sequences for detecting genes by RTq-PCR.

Gene		Primer sequence
Other-14:31-tRNA-Gly-CCC-3	F primer	5’-TGGCGGACGAATGGTAGAAT-3’
	R primer	5’-TATCCTTGTTCACGACTCCTTCAC-3’
Other-39:73-tRNA-Arg-TCG-5	F primer	5’-TCAGAAGATTGAGGGTTCGAATC-3’
	R primer	5’-GTGCAGGGTCCGAGGT-3’
U6	F primer	5’-ATTGGAACGATACAGAGAAGATT-3’
	R primer	5’-GGAACGCTTCACGAATTTG-3’

### 2.6 Gene ontology and pathway enrichment analysis

Gene ontology (GO) and Kyoto Encyclopedia of Genes and Genomes (KEGG) pathway enrichment analysis for the differentially expressed tsRNAs was conducted using the Database for Annotation, Visualization and Integrated Discovery (DAVID) v6.8 online tool. GO analysis categorizes genes into three domains: biological process (BP), molecular function (MF), and cellular component (CC). This allowed functional annotation of the tsRNAs and prediction of their biological roles. KEGG analysis identifies enriched metabolic or signaling pathways among groups of genes, providing insights into their biological interactions and functions. A modified Fisher’s exact test was used to determine enrichment, and Benjamini-Hochberg method to adjust p-values for multiple testing. GO terms and pathways with adjusted p-values < 0.05 were considered significantly enriched. The results of GO and KEGG enrichment analysis facilitated characterization of the functional roles and mechanisms of action of tsRNA dysregulated in POD.

### 2.7 Local target prediction from sequence

This target prediction method integrates two algorithms: the first is miRanda, a classic dynamic programming algorithm that can identify any Seed-type site based on RNA secondary structure and free energy (30). The second is TargetScan, which combines mRNA and miRNA expression profile data and uses a fitting method to find biologically significant site sequence features and a scoring model with relative conservation, capable of searching for perfect match sites of 8mer, 7mer-m8, and 7mer-1a with 2-6, 2-7, and 2-8 nucleotides (31–33). By combining these two algorithms, not only their respective advantages are utilized, but also the results are improved, making target prediction more accurate and easier to interpret.

## 3 Statistical analysis

Statistical analysis was performed using SPSS Statistics software version 26.0 (IBM Corp., Armonk, NY, USA). Normality test was performed on all measurement data in this study, and the statistical description of continuous measurement data (such as age, BMI, hemoglobin, CRP, etc.) conforming to normal distribution was adopted the mean ± standard (X ± SD), and independent sample t test was used for inter-group comparison. The measurement data of non-normal scores were represented by the median (upper quartile and lower quartile) [M(Q1, Q3)], and non-parametric independent sample Mann-Whitney U test was used for comparison between groups. Statistical descriptions of disordered statistical data (such as sex, hypertension, diabetes, coronary atherosclerotic heart disease, etc.) were expressed in the form of frequency and percentage, and comparison of frequency distribution differences between the two groups was analyzed using χ^2^ test or Fisher exact probability method. A paired t-test was used to compare pre-and postoperative plasma tsRNAs levels within each group. Receiver operating characteristic (ROC) curve analysis was conducted to evaluate the diagnostic performance of plasma tsRNAs. Two-tailed p-values <0.05 were considered statistically significant. GraphPad Prism 8 software was used for graphing and figures.

To control for potential confounding from baseline differences between the POD and non-POD groups, we performed propensity score matching (PSM). This statistical technique aims to balance measured covariates between comparator groups to simulate a randomized controlled trial design. The POD group and the Non-POD group were matched using STATA software with a caliper value of 0.05. The covariates used for matching included age, ASA classification, operation duration, operation type, and anesthesia method. After 1:1 nearest neighbor matching, the POD group and the Non-POD group with good balance between groups were obtained. PSM effectively controlled the baseline differences, simulated a randomized controlled trial, and made the experiment more valid both internally and externally.

## 4 Results

### 4.1 High-throughput sequencing of tsnRNA in plasma of elderly POD patients

In this study, the plasma samples of 4 postoperative normal elderly patients (CON-1, CON-2, CON-3, CON-4) and 4 POD patients (POD-1, POD-2, POD-3, POD-4) were selected for high-throughput sequencing. In order to reduce the influence of other factors, We selected the plasma of 4 postoperative normal patients with similar clinical data to the 4 POD patients, including gender, age, ASA grade, type of surgery, years of education, and related comorbidities. The sample information of the two groups is shown in **Table 2**.

**Table 2:** Sample information of POD patients and postoperative normal patients.

Group	Gender	Age	ASA	Degree of education	Type of operation
CON-1	Female	81	II	Primary school	Hip replacement
CON-2	Female	76	III	Primary school	Femoral fracture internal fixation
CON-3	Male	85	II	Junior high school	Hip replacement
CON-3	Male	72	III	Senior high school	Knee replacement
POD-1	Female	80	II	Primary school	Hip replacement
POD-2	Female	77	III	Primary school	Femoral fracture internal fixation
POD-3	Male	85	II	Junior high school	Hip replacement
POD-4	Male	72	III	Senior high school	Knee replacement

### 4.2 Differential expression of tsRNA in plasma of POD patients

**Figure 2:**
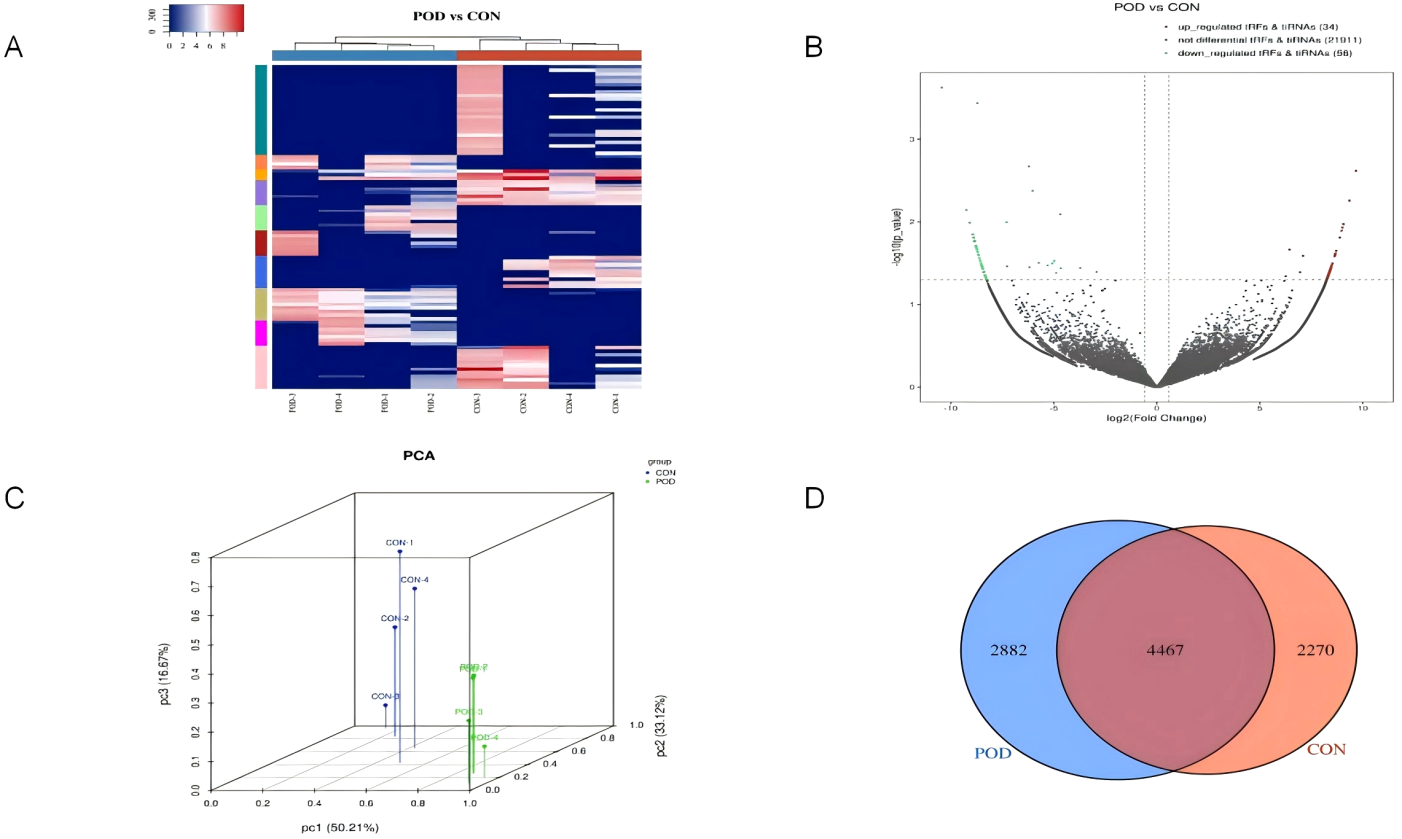
Differentially expressed genes for tsRNA levels in plasma of Non-POD patients and POD patients after surgery. **(A)** The tsRNA expression profiles in postoperative delirium patients were analyzed using unsupervised hierarchical cluster heat maps of tsRNA. The expression levels were represented by different colors on the heat map, ranging from blue (below average) to red (above average). The samples were categorized and separated using K-means clustering. **(B)** Volcanic maps were utilized to compare the expression levels of tsRNA between the two groups. The X-axis typically depicts the variation in gene expression levels. On the other hand, the Y-axis illustrates the statistical significance of said variation. Upregulated tsRNA were denoted by red dots above the red line, while down-regulated tsRNA were indicated by green dots below the red line. Non-differentially expressed tsRNA were represented by gray dots. **(C)** To determine the main factors influencing the expression level of the samples, Principal component analysis was conducted. The X, Y, and Z axes represented these factors, while the colored dots corresponded to the samples. The position of each point represented the primary characteristics of the sample, and the distance between the points reflected the similarity in data size. **(D)** A Venn diagram was employed to compare the quantity of commonly expressed and specifically expressed tsRNA between the two groups. This diagram illustrated the overlap and unique expression patterns.

The cluster analysis diagram provides a visual representation of the expression patterns of tsRNA in patients experiencing POD compared to those who are postoperative normal patients (**Figure 2A**). This analytical diagram integrates both sample clustering and gene clustering methodologies, thereby facilitating the rapid identification of expression similarities among various samples and genes. Each column in the diagram corresponds to a specific sample, with samples clustered based on the similarity of gene expression profiles, allowing for the aggregation of similar samples. Each row denotes a tsRNA gene, with the organization of genes predicated upon their expression similarities across the sampled data. Through the process of clustering, genes exhibiting analogous expression patterns are systematically grouped. The gradient of color utilized in the diagram indicates the abundance or level of gene expression, where red typically signifies up-regulated gene expression, in contrast to blue which signifies down-regulated gene expression. The heat map derived from cluster analysis unequivocally illustrates the pronounced disparities in expression patterns observed between POD patients and postoperative normal patients. The volcano plot representing the differential expression of tsRNA conveys both the expression fold change (FC) of tsRNA and the statistical significance of these variations simultaneously, thereby enabling the direct identification of up-regulated and down-regulated tsRNA, with 34 tsRNAs being up-regulated (depicted in red). Conversely, the analysis revealed that 56 tsRNAs were down-regulated (illustrated in green), while those exhibiting no significant differential expression between the two patient cohorts are represented in gray (**Figure 2B**). Principal Component Analysis (PCA) serves as a robust statistical technique for unsupervised analysis aimed at the dimensionality reduction of extensive datasets, proving to be an invaluable tool for the examination of sample classifications based on expression data. By employing PCA, the original tsRNA expression dataset, which may encompass a multitude of variables, can be distilled into a limited number of principal components, each representing a substantial fraction of the variation inherent in the original dataset. The positioning of each sample within the principal component score chart correlates with its respective score across each principal component. Consequently, an examination of the sample distribution on the score plot enables the visual detection of distinctions in tsRNA expression patterns between POD patients and postoperative normal patients (**Figure 2C**). The discernible tsRNA expression patterns distinguishing POD patients from postoperative normal patients have been effectively visualized through principal component analysis. The Venn diagram serves to elucidate the overlap of differential expression of tsRNA between POD patients and postoperative normal patients, visually depicting the quantity of tsRNA co-expressed between the two groups, while also delineating the amount of tsRNA that is uniquely expressed within each group (**Figure 2D**). Specifically, 4467 tsRNAs were found to be co-expressed in both cohorts, while 2882 tsRNAs were uniquely expressed in POD patients, and 2270 tsRNAs were exclusively expressed in postoperative normal patients.

### 4.3 Selection of candidate genes

In order to delineate pivotal differentially expressed genes necessitating subsequent investigation, this research employed stringent selection criteria pertaining to the sequencing data with the objective of prioritizing targets exhibiting the most compelling evidence of biological and clinical relevance in relation to the study’s hypotheses. Initially, the R package edgeR was utilized to evaluate the differentially expressed tsRNAs. Based on the thresholds of FC > 1.5 and P < 0.05, a total of 90 significantly differentially expressed tsRNAs were identified, 34 of which were notably upregulated. Conversely, 56 demonstrated downregulation. This investigation concentrated on genes exhibiting the most pronounced alterations in expression levels between the two groups, as these genes are posited to have a direct impact on the observed phenotypic manifestations. Ultimately, Other-14:31-tRNA-Gly-CCC-3 and Other-39:73-tRNA-Arg-TCG-5 were designated as candidate genes (**Table 3**).

**Table 3:** Data file content of the significantly differentially expressed tsRNA.

tsRNA	Length	Fold_Change	Regulation	*P*
Other-14:31-tRNA-Gly-CCC-3	18	812.9835508	Up	0.002416916
Other-39:73-tRNA-Arg-TCG-5	35	516.0845998	Up	0.011743269

### 4.4 Patient characteristics

**Figure 3:**
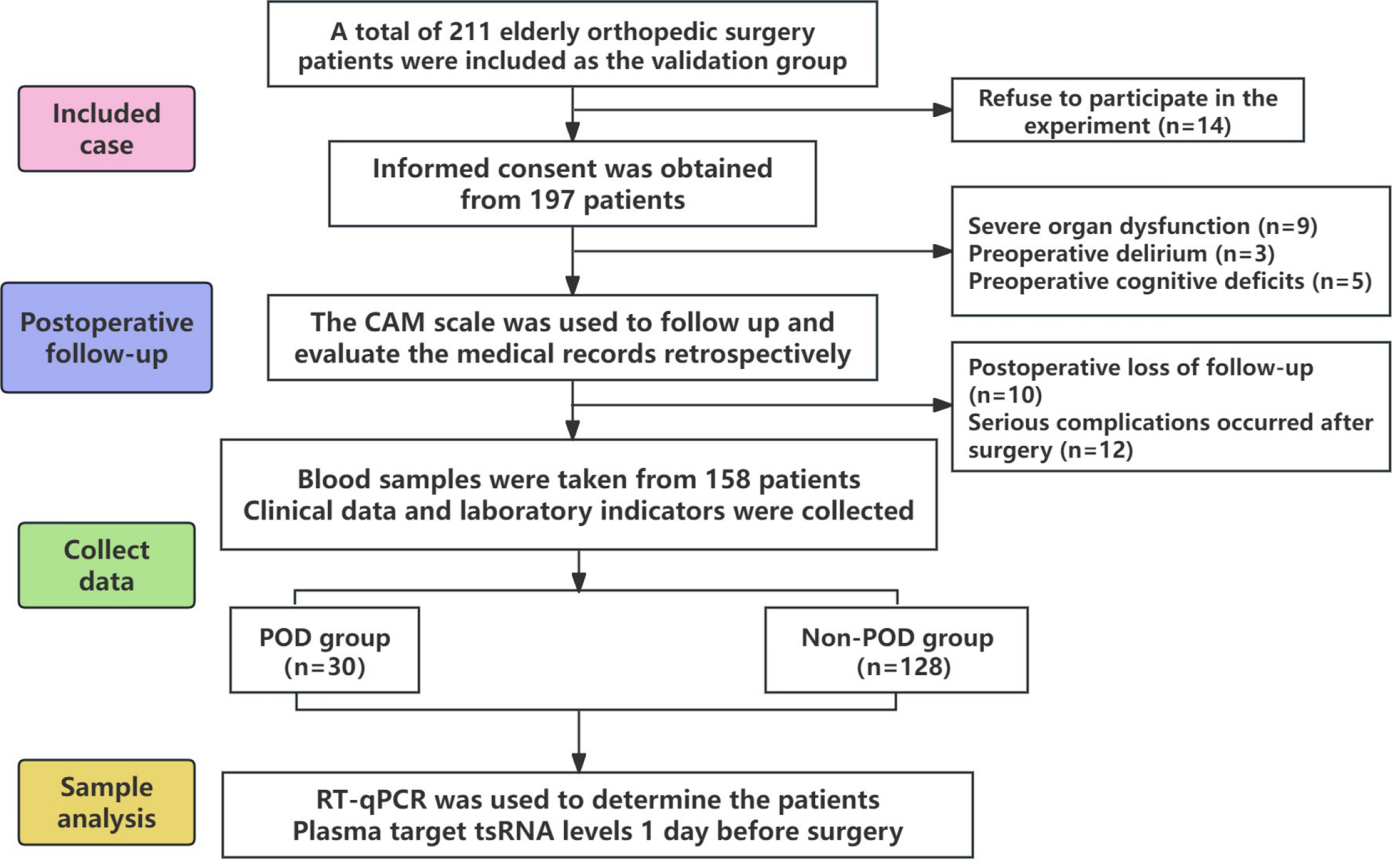
Patient recruitment flow chart.

This investigation encompassed a cohort of 158 geriatric patients who underwent orthopedic surgical procedures, comprising 30 individuals within the POD group and 128 individuals within the Non-POD group. **Table 4** presents a comparative analysis of the demographic and clinical attributes of the POD and Non-POD cohorts. The average age of the POD cohort was markedly elevated when juxtaposed with that of the Non-POD cohort (79.07 ± 6.54 vs 73.35 ± 7.56, P < 0.001). The gender distribution exhibited no statistically significant difference between the two cohorts (P = 0.763), nor was there a significant disparity in educational attainment between the two cohorts (P = 0.370). In comparison to the postoperative normal cohort, the proportion of patients classified with ASA grade III in the POD cohort was significantly greater (P = 0.014). Furthermore, individuals within the POD cohort demonstrated a lower mean BMI than their Non-POD counterparts (21.97 ± 4.04 vs 23.94 ± 3.78 kg/m², P = 0.026). There were no notable differences in the prevalence of hypertension, coronary artery disease, hyperlipidemia, or cerebrovascular disease across all groups. Regarding surgical factors, the procedures involving open reduction and internal fixation of femoral fractures were significantly correlated with the occurrence of POD when compared to total hip or knee replacements (P = 0.009). The utilization of general anesthesia was likewise associated with an augmented incidence of POD in comparison to subarachnoid anesthesia or combined regional nerve block approaches (P = 0.009). The mean duration of surgical procedures in the POD cohort was significantly prolonged relative to that of the Non-POD cohort (P = 0.027). In contrast to the Non-POD cohort, the POD cohort experienced a lengthier postoperative hospital stay, a heightened rate of ICU admission, and an elevated postoperative VAS score (P < 0.001). In conclusion, these data suggest that advanced age, ASA class III designation, reduced BMI, type of surgical intervention, general anesthesia usage, extended surgical duration, postoperative ICU occupancy, and elevated VAS pain scores are significantly associated with the incidence of POD. Other demographic factors and comorbid conditions did not reveal a significant association. The patient recruitment flow chart is shown in **Figure 3**.

**Table 4:** Demographics, clinicopathological characteristics associated with POD in elderly patients.

Patient characteristics	POD		*P*
	Yes(n=30)	No(n=128)	
Age (year)	79.07 ± 6.54	73.35 ± 7.56	0.000***
Gender, n (%)			0.763
Male	13(43.3%)	58(45.3%)	
Female	17(56.7%)	70(54.7%)	
MMSE	25.08 ± 3.28	26.43 ± 2.96	0.217
ASA physical status, n (%)			0.014*
II	10(33.3%)	75(58.6%)	
III	20(66.7%)	53(41.4%)	
BMI (kg/m2)	21.97 ± 4.04	23.94 ± 3.78	0.026*
Mate			0.066
Alive	19(63.3%)	90(70.3%)	
Death	11(36.7%)	38(29.7%)	
Comorbidities, n (%)			
Hypertension	15(50.0%)	47(36.7%)	0.419
Diabetes	4(13.3%)	15(11.7%)	0.041*
Coronary heart disease	7(23.3%)	11(8.5%)	0.116
Hyperlipidemia	4(13.3%)	21(16.4%)	0.26
Cerebrovascular disease personal history	6(20.0%)	14(10.9%)	0.063
Types of surgery, n (%)			0.009*
Total hip replacement	12(40.0%)	56(43.7%)	
Total knee replacement	3(10.0%)	42(32.8%)	
Open reduction and internal fixation of femoral fracture	15(50.0%)	30(23.4%)	
Mode of anesthesia			0.009*
General anesthesia	21(70.0%)	18(14.1%)	
Subarachnoid anesthesia	8(26.7%)	87(67.9%)	
General anesthesia combined with nerve block	1(3.30%)	23(18.0%)	
Operation duration	2[1-7]	1.5[1-5]	0.027*
Estimated blood loss (ml)	200[50-800]	100[30-600]	0.101
Length of stay	19[9-25]	12[3-21]	0.023*
ICU administration	4[2-5]	3[1-6]	0.000***

POD, postoperative delirium; ASA, American Society of Anesthesiologists; BMI, Body Mass Index. *P value<0.05 by Chi-square test, Fisher exact test, t test or Mann Whitney U test, **P < 0.01, ***P < 0.001.Mean ± standard deviation (X ± SD) was employed for normal continuous data description, with independent sample t test for group comparisons. Non-normal measurement data were represented by the median [M(Q1, Q3)], utilizing the non-parametric Mann-Whitney U test for group comparisons.

### 4.5 Preoperative laboratory tests and POD

Preoperative laboratory assessments were systematically analyzed and juxtaposed between the POD cohort and the Non-POD cohort (**Table 5**). The preoperative hemoglobin concentration within the POD cohort was statistically significantly inferior to that observed in the Non-POD cohort (111.24 ± 23.91 vs 126.96 ± 20.65 mg/dL, P = 0.001). Furthermore, the preoperative CRP levels recorded in the POD cohort were markedly elevated compared to those in the Non-POD cohort (25.22 ± 45.55 vs 12.82 ± 25.89 mg/dL, P = 0.032). The lymphocyte enumeration in the POD cohort was significantly diminished relative to the Non-POD cohort (1.02 ± 0.44 vs 1.44 ± 0.55 × 10^9/L, P < 0.001). In a similar vein, the preoperative plasma albumin concentration within the POD cohort was significantly lower than that in the Non-POD cohort (35.54 ± 4.90 vs 39.30 ± 4.38 g/L, P < 0.001). The preoperative D-dimer concentrations in the POD cohort were significantly higher than those in the Non-POD cohort (2051.56 ± 2713.097 vs 751.12 ± 1214.85 mg/L, P = 0.001). Notably, there were no statistically significant variations in preoperative platelet counts, neutrophil counts, or creatinine levels across all cohorts. In conclusion, based on these findings, diminished preoperative hemoglobin levels, elevated CRP levels, reduced lymphocyte counts, lower albumin levels, and increased D-dimer concentrations were correlated with an augmented risk of POD.

**Table 5:** Preoperative laboratory tests associated with POD in elderly patients.

Preoperative laboratory tests	POD		P
	Yes (n=27)	No (n=63)	
Hemoglobin (mg/dL)	111.24 ± 23.91	126.96 ± 20.65	0.001***
Platelet (x10^9^/L)	229.10 ± 82.33	213.63 ± 60.84	0.288
CRP (mg/dL)	25.22 ± 45.55	12.82 ± 25.89	0.032*
Neutrophil count (x10^9^/L)	6.14 ± 3.45	4.89 ± 2.29	0.078
Lymphocyte count (x10^9^/L)	1.02 ± 0.44	1.44 ± 0.55	0.000***
Creatinine (μmol/L )	89.87 ± 80.16	69.88 ± 20.08	0.058
Albumin (g/L)	35.54 ± 4.90	39.30 ± 4.38	0.000***
D-dimer (mg/L)	2051.56 ± 2713.097	751.12 ± 1214.85	0.001***

POD, postoperative delirium; CRP, C-reactive protein. *P value<0.05 by t test or Mann Whitney U test.*P < 0.05, **P < 0.01, ***P < 0.001.Mean ± standard deviation (X ± SD) was employed for normal continuous data description, with independent sample t test for group comparisons. Non-normal measurement data were represented by the median [M(Q1, Q3)], utilizing the non-parametric Mann-Whitney U test for group comparisons.

### 4.6 Serum expressions of tsRNAs and POD

**Figure 4:**
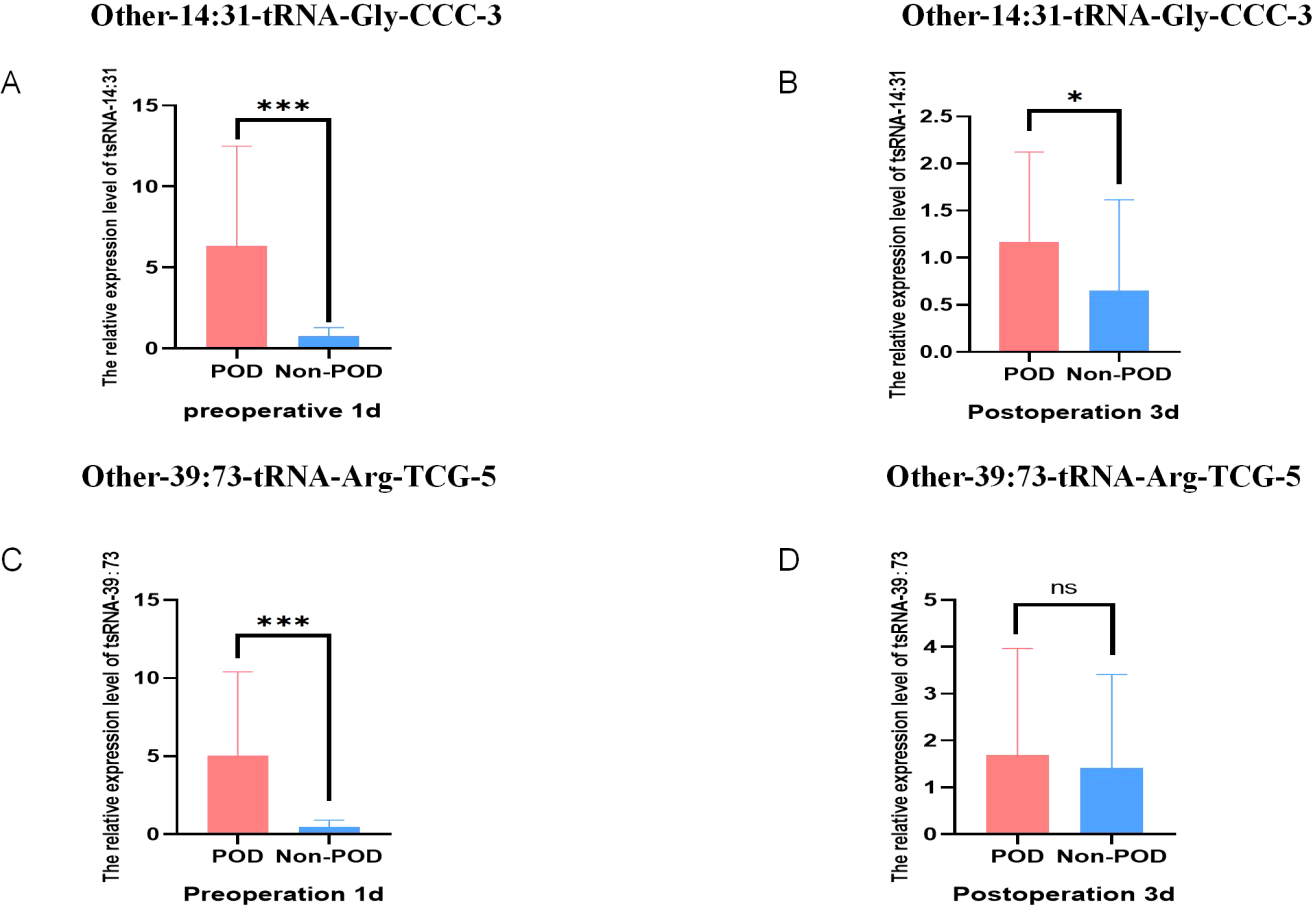
Relative expression of POD-related tsRNA in peripheral blood of elderly patients. **(A)** The relative level of Other-14:31-tRNA-Gly-CCC-3 in RT-qPCR results of Non-POD group and POD group 1 day before surgery; **(B)** The relative level of Other-14:31-tRNA-Gly-CCC-3 in the RT-qPCR results of the Non-POD group and the POD group 3 day after surgery; **(C)** The relative level of Other-39:73-tRNA-Arg-TCG-5 in RT-qPCR results of Non-POD group and POD group 1 day before surgery; **(D)** The relative level of Other-39:73-tRNA-Arg-TCG-5 in RT-qPCR results of Non- POD group and POD group 3 day after surgery. *P<0.05, ***P<0.001, ns means not significant.

A total of 158 geriatric patients undergoing orthopedic surgery who satisfied the stipulated inclusion criteria were categorized into a 30 POD group and a 128 Non-POD group based on the CAM scale. Comprehensive RNA extraction was performed on the plasma samples obtained one day prior to the surgical procedure and three days post-operation, followed by the synthesis of total RNA from the patients into complementary DNA (cDNA) through reverse transcription methods. Subsequently, the RT-qPCR technique was employed to ascertain the expression levels of the aforementioned two target small RNA species in these samples, utilizing U6 as the internal control reference. The outcomes of the RT-qPCR analysis revealed that, in comparison to the control cohort, the expression levels of these two target tsRNAs were markedly elevated in the POD group one day prior to surgery (P < 0.001) (**Figures 4A, C**). The expression level of Other-14: 31-tRNA-Gly-CCC-3 in the POD group remained significantly higher than that observed in the Non-POD group three days following the surgical intervention (P=0.046), whereas Other-39: 73-tRNA-Arg-TCG-5 exhibited no statistically significant variance between the POD group and the Non-POD group three days post-operation (P = 0.584) (**Figures 4B, D**). In summary, the findings suggest that the elevation of plasma Other-14: 31-tRNA-Gly-CCC-3 and Other-39: 73-tRNA-Arg-TCG-5 levels one day preceding the surgical procedure may be associated with the development of POD in patients.

### 4.7 Predictive value of tsRNAs for POD

**Figure 5:**
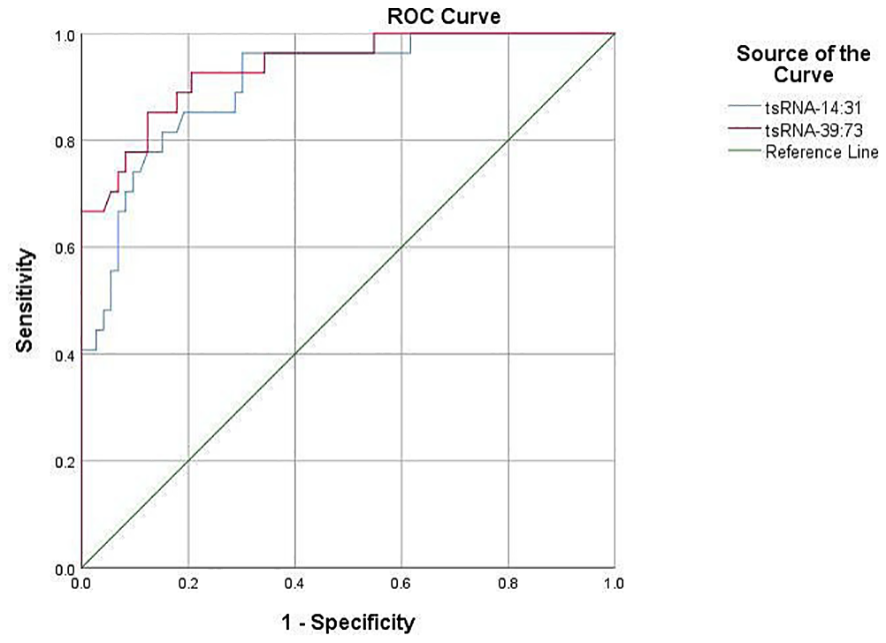
Diagnostic value of target tsRNA as a biomarker in POD.

The predictive value of Other-14:31-tRNA-Gly-CCC-3 and Other-39:73-tRNA-Arg-TCG-5 in forecasting POD was systematically assessed utilizing the Receiver Operating Characteristic (ROC) curve. The area under the ROC curve serves as a pivotal metric for evaluating the diagnostic efficacy of test indicators, wherein a larger value correlates with enhanced predictive capability. For Other-14:31-tRNA-Gly-CCC-3, the area under the ROC curve achieved a value of 0.868, signifying its effectiveness in accurately identifying patients at risk for POD. At the optimal threshold of 0.435, the sensitivity for predicting POD was determined to be 81.5%, while the specificity reached 86.3%. This finding robustly illustrates the considerable potential of Other-14:31-tRNA-Gly-CCC-3 in POD prediction. Concurrently, an ROC analysis was conducted for Other-39:73-tRNA-Arg-TCG-5. The findings revealed that the area under the curve was 0.936, which unequivocally satisfied the criteria for a favorable predictive level, thereby substantiating its diagnostic capability. At the critical threshold of 0.355, the sensitivity and specificity for predicting POD were reported at 92.6% and 91.8%, respectively, indicating a high degree of accuracy. Collectively, Other-14:31-tRNA-Gly-CCC-3 and Other-39:73-tRNA-Arg-TCG-5 exhibited formidable predictive power in ROC analysis, with their area under the curve and elevated sensitivity demonstrating an adeptness in differentiating delirious patients from their non-delirious counterparts (**Figure 5**). These findings distinctly suggest that preoperative plasma levels of OTHER-14:31-tRNA-Gly-CCC-3 and Other-39:73-tRNA-Arg-TCG-5 may serve as robust predictive biomarkers for the occurrence of POD in elderly patients undergoing orthopedic surgery. Such insights will be instrumental for the clinical advancement of risk assessment and decision-making processes.

The ROC curve was employed to evaluate the comparative expression levels of the two targeted tsRNAs within the predicted POD of geriatric patients.Other-14:31-tRNA-Gly-CCC-3was a predictor of POD before surgery, with an AUC of 0.868 (*P*< 0.001). Serum Other-39: 73-tRNA-Arg- TCG-5 was also a significant predictor of POD, with an AUC of 0.936 (*P* < 0.001).

### 4.8 Propensity score matching analysis and adjustment

**Figure 6:**
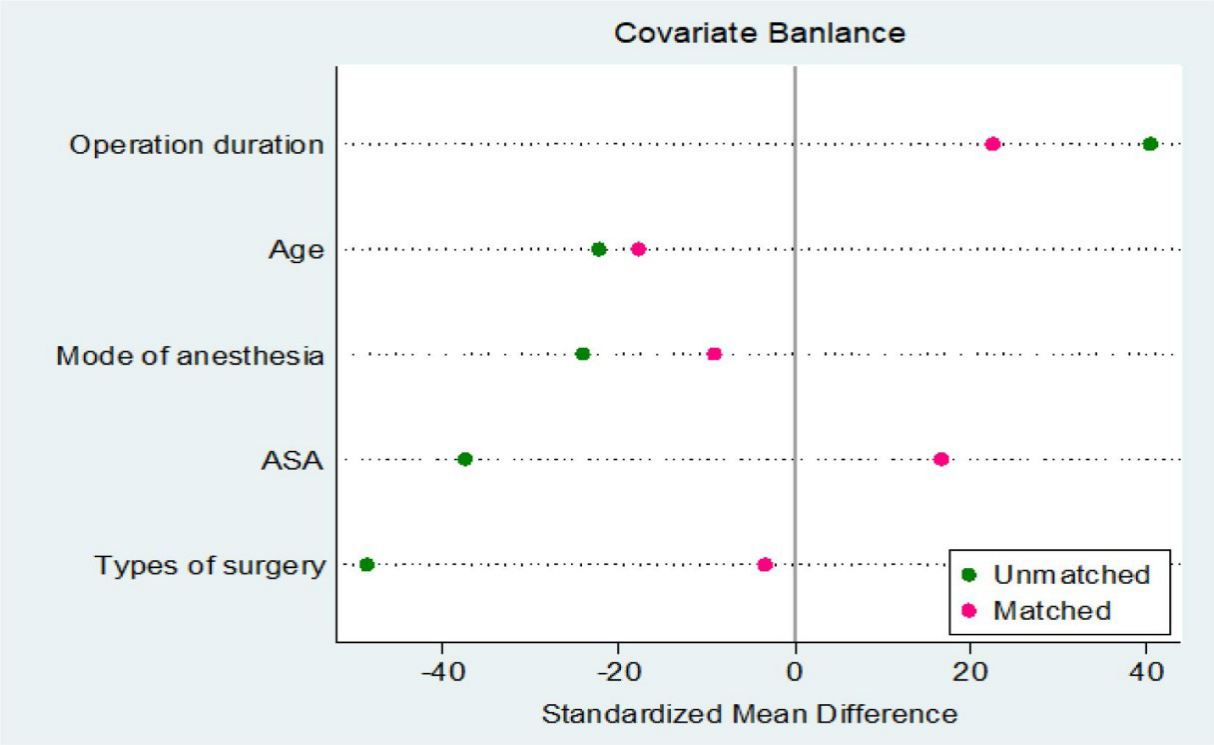
Plot of the standardized mean differences for the estimated propensity scores (distance) and the matching variables.

Since the differences in baseline characteristics between POD group and Non-POD group may introduce confounding, we further adjusted the two groups by using PSM method. This method calculated the probability of POD occurrence for each patient and performed a 1:1 match between the POD and Non-POD groups to balance the differences between the two groups in age, ASA grade, duration of surgery, type of surgery, and method of anesthesia. In this study, the Nearest Neighbor Matching method is used to carry out covariate matching, which is the most frequently used matching strategy among PSM methods. The pre-calculated propensity score is used to search for individuals with the smallest difference in propensity score in the control group according to the scores of individuals in the experimental group. In this way, each POD group individual can find a most matched Non-POD pairing object, so as to realize the matching of the two groups of individuals in the tendency characteristics, which has the advantage of more full use of information, which is conducive to improving the matching degree and reducing the influence of selection bias on the result. Therefore, 29 balanced sample pairs are finally formed for subsequent research. **Figure 6** shows the sample distribution after PSM. By matching PSM, POD group and Non-POD group were successfully balanced in baseline covariates, eliminating their natural differential effects. From **Figure 6**, we can see that the application of PSM in this study reliably controls confounding factors and provides a balanced sample for subsequent analysis.

The data points of 29 individuals in the POD cohort and 29 individuals in the control cohort were dispersed in the figure. Following the matching process, a significant portion of the data points congregated around the optimal regression line, suggesting a close alignment in baseline characteristics between the two cohorts concerning these two parameters. Despite some individual data points deviating from the linear fit, the overall distribution of data points appears homogeneous, with no evident clustering tendency, signifying the successful attainment of an ideal matching effect.

### 4.9 Analysis of relative expression levels of target tsRNA in plasma after PSM

**Figure 7:**
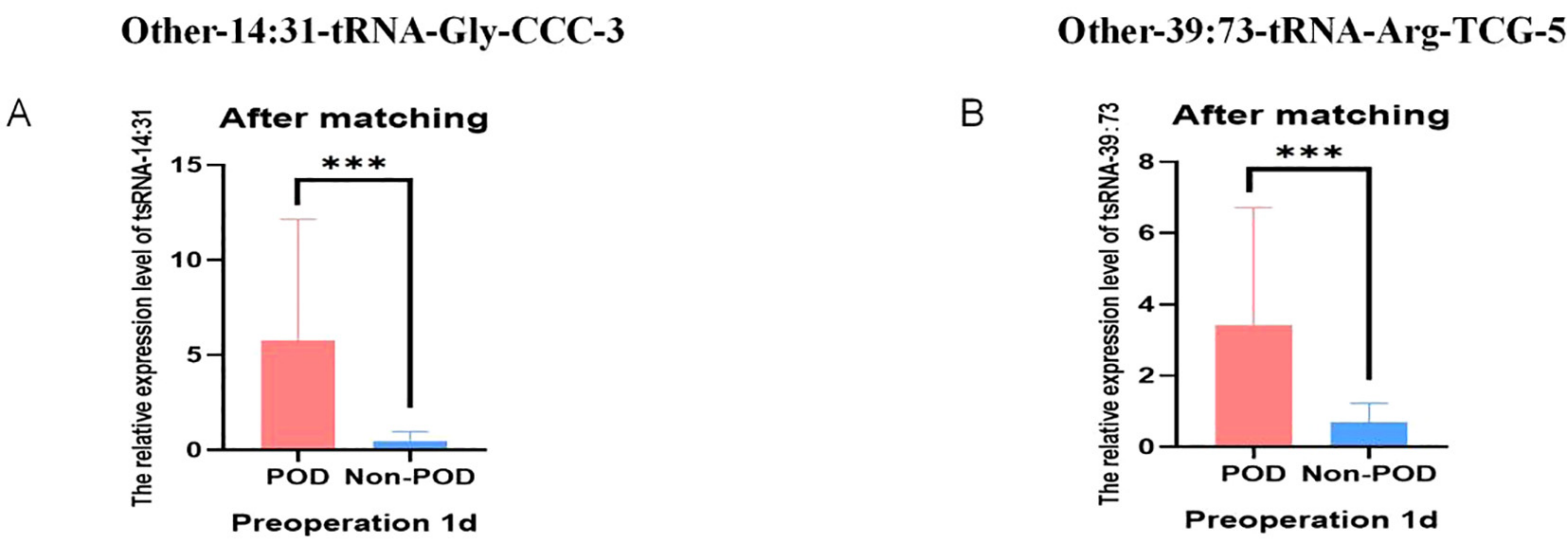
Relative expression of POD-associated tsRNA in peripheral blood of elderly patients after PSM. **(A)** The relative levels of Other-14:31-tRNA-Gly-CCC-3 of Non-POD and POD groups from RT-qPCR after PSM; **(B)** The relative levels of Other-39:73-tRNA-Arg-TCG-5 of Non-POD and POD groups from RT-qPCR after PSM. ***P<0.001.

To test the ability of Other-14:31-tRNA-Gly-CCC-3 and Other-39:73-tRNA-Arg-TCG-5 to predict POD, we analyzed the results obtained before and after pairing, respectively. **Figures 4A, B** before PSM showed that the expression levels of Other-14:31-tRNA-Gly-CCC-3 and Other-39:73-tRNA-Arg-TCG-5 in the POD group in the verification cohort were significantly increased. Validation in the cohort after PSM also reached the same conclusion. In addition, we further tested this finding using 29 pairs of matched and balanced samples. **Figures 7A, B** show that after controlling confounding factors, Other-14:31-tRNA-Gly-CCC-3 and Other-39: The expression level of 73-tRNA-Arg-TCG-5 in POD group was still significantly higher than that in control group (*P* < 0.001), confirming that they were reliable predictors of POD occurrence.

**Figure 8:**
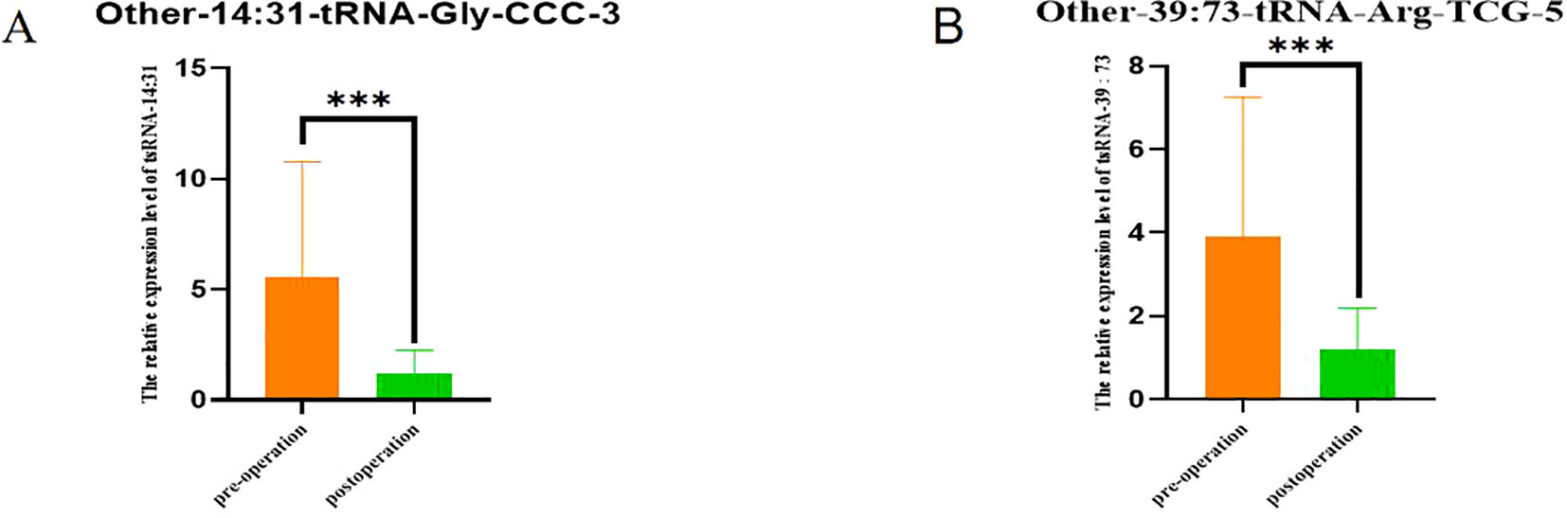
Comparison of tsRNA expression levels in POD group 1 day before surgery and 3 days after surgery. **(A)** Comparison of expression levels of Other-14:31-tRNA-Gly-CCC-3 in POD group 1 day before surgery and 3 days after surgery; **(B)** Comparison of expression levels of Other-39:73-tRNA-Arg-TCG-5 in POD group 1 day before surgery and 3 days after surgery. ***P<0.001.

The **Figure 8** compared the expression changes of these two tsRNA at two time points before and after surgery, and the ordinate was the difference of target tsRNA expression at the two time points. In **Figure 8A**, for Other-14:31-tRNA-Gly-CCC-3, there was a significant difference in expression level between the Non-POD group and the POD group (P<0.001). Similarly, for Other-39:73-tRNA-Arg-TCG-5, there were also significant differences in expression levels between the two groups (P<0.001). These results suggest that changes in the expression of these two tsRNAs before and after surgery are more pronounced in POD patients.

**Figure 9:**
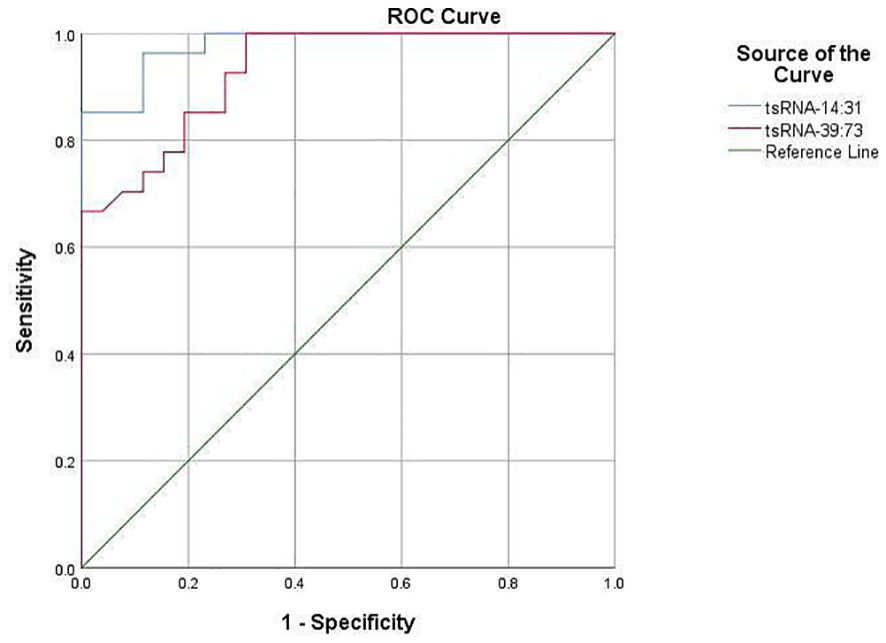
The diagnostic value of target tsRNA as a marker in POD after PSM.

In the PSM cohort, subsequent validation post-matching demonstrated that Other-14:31-tRNA-Gly-CCC-3 and Other-39:73-tRNA-Arg-TCG-5 remained robust indicators of POD, exhibiting AUC values of 0.931 and 0.979, respectively (**Figure 9**). Collectively, PSM adeptly mitigated baseline disparities and further substantiated the independence of Other-14:31-tRNA-Gly-CCC-3 and Other-39:73-tRNA-Arg-TCG-5 in forecasting POD.

The ROC curve was utilized to analyze the relative expression of serum Other-14: 31-tRNA-Gly-CCC-3and Other-39: 73 tRNA-Arg-TCG-5 before and after PSM in predicting POD in elderly patients. Other-14:31-tRNA-Gly-CCC-3 was a predictor of POD before surgery, with an AUC of 0.931 (P < 0.001). Serum Other-39: 73 -tRNA-Arg-TCG-5 was also an important predictor of POD, with an AUC of 0.979(*P* < 0.001).

### 4.10 Logistic regression analysis of related factors in POD patients before operation

This study takes the occurrence of POD as the dependent variable, and takes the laboratory examination indicators of patients such as hemoglobin, CRP, lymphocyte count, albumin, D-dimer, and two target tsRNAs, a total of 7 factors, as covariates, to conduct a preliminary analysis of the risk factors related to POD.

After the initial univariate binary Logistic regression analysis, it was found that the following four factors were significantly associated with POD (**Table 6**): patients with high expression of Other-14: 31-tRNA-Gly-CCC-3 before surgery had an increased risk of POD (OR = 3.455, 95% CI: 1.613 - 7.400); patients with elevated expression levels of Other-39: 73-tRNA-Arg-TCG-5 before surgery had an increased risk of POD (OR = 6.081, 95% CI: 2.832 - 22.913); patients with lower preoperative lymphocyte counts had a higher risk of POD (OR = 0.229, 95% CI: 0.064 - 0.822); and patients with lower preoperative albumin levels were more prone to POD (OR = 0.835, 95% CI: 0.719 - 0.971).

**Table 6:** Univariate logistic regression analysis of influencing factors in POD patients.

Influencing factor	OR	95%CI	*P*
Other-14: 31-tRNA-Gly-CCC-3	3.455	1.613-7.400	0.001***
Other-39: 73-tRNA-Arg-TCG-5	6.081	2.832-22.913	0.000***
hemoglobin	0.966	0.966-1.027	0.819
CRP	0.998	0.982-1.015	0.814
lymphocyte count	0.229	0.064-0.822	0.024*
albumin	0.835	0.719-0.971	0.019*
D-dimer	1.001	1.000-1.001	0.059

*P<0.05, ***P<0.001.

Taking whether the subjects developed POD as the dependent variable, the four initially screened factors were further included as covariates in a multivariate binary logistic regression model for regression analysis (**Table 7**). The results indicated that patients with elevated preoperative expression levels of Other-14:31-tRNA-Gly-CCC-3 had an increased risk of POD (OR = 0.117, 95% CI: 0.019 - 0.71); and patients with elevated preoperative expression levels of Other-39:73-tRNA-Arg-TCG-5 also had an increased risk of POD (OR = 0.098, 95% CI: 0.023 - 0.409).

**Table 7:** Multivariable logistic regression analysis of influencing factors in POD patients.

Influencing factor	OR	95%CI	*P*
Other-14:31-tRNA-Gly-CCC-3	0.117	0.019-0.71	0.002**
Other-39:73-tRNA-Arg-TCG-5	0.098	0.023-0.409	0.001**
lymphocyte count	5.631	0.211-149.946	0.302
albumin	1.375	0.913-2.070	0.128

**P<0.01.

In conclusion, the high expression of Other-14:31-tRNA-Gly-CCC-3 and Other-39: 73-tRNA-Arg-TCG-5 before surgery were independent risk factors for POD.

### 4.11 Gene ontology and pathway enrichment analysis

The current study used gene ontology (GO) and KEGG pathway enrichment analysis to gain insights into the functions of dysregulated tsRNAs in postoperative delirium.

**Figure 10:**
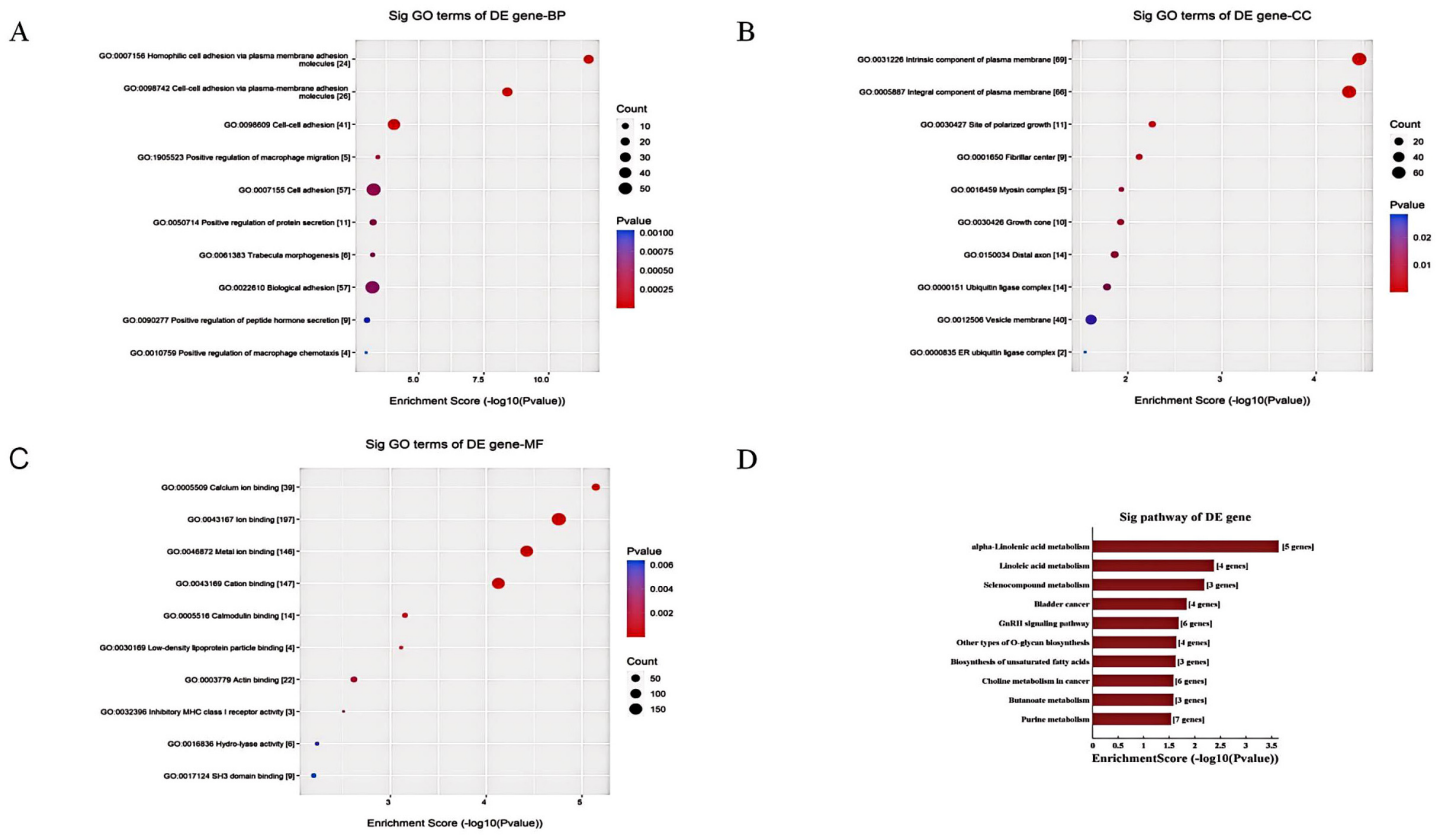
The GO and KEGG pathway enrichment analysis of dysregulated tsRNAs in POD. **(A)** The BP annotation information of differentially expressed tsRNAs in POD and non-POD group; **(B)** The CC annotation information of differentially expressed tsRNAs in POD and non-POD group; **(C)** The MF annotation information of differentially expressed tsRNAs in POD and non-POD group; **(D)** Kyoto Encyclopedia of Genes and Genomes pathway analyze of differentially expressed tsRNAs in POD and non-POD group.

GO analysis revealed enrichment of terms related to biological processes, cellular components, and molecular functions. The top enriched terms for biological processes were related to inflammatory response, leukocyte activation, and ion transport (**Figure 10A**), suggesting roles in neuroinflammation and neuronal excitability. In cellular component analysis, synaptic and neuronal projection domains were significantly enriched (**Figure 10B**), indicating potential localization of target genes in cognition-related structures. At the molecular level, ion binding and enzyme regulator activity terms were prominently enriched (**Figure 10C**), suggesting modulation of protein functions involved in neurotransmission.

KEGG pathway analysis identified overrepresented pathways associated with cognition, including neuroactive ligand-receptor interaction and calcium signaling pathways. Impaired key functions in delirium, such as glutamatergic and cholinergic synapses, were also identified. Enriched metabolism pathways included linoleic acid metabolism, purine metabolism, and selenocompound metabolism. Linoleic acid, an omega-3/6 fatty acid, impacts neuronal integrity and pathology. Purines modulate neurotransmission, metabolism, and blood flow. Selenoproteins influence redox homeostasis (**Figure 10D**).

In conclusion, the GO and KEGG analyses suggest that dysregulated tsRNAs play roles in ion binding, neurosignaling, neuroinflammation, immune responses, lipid and compound metabolism, and amino acid metabolism, all of which are important for optimal brain function. These findings indicate that disturbances in tsRNAs may contribute to delirium by affecting core neuronal, metabolic, and inflammatory pathways through targeted gene regulation.

### 4.12 Targeted tsRNAs network construction

**Figure 11:**
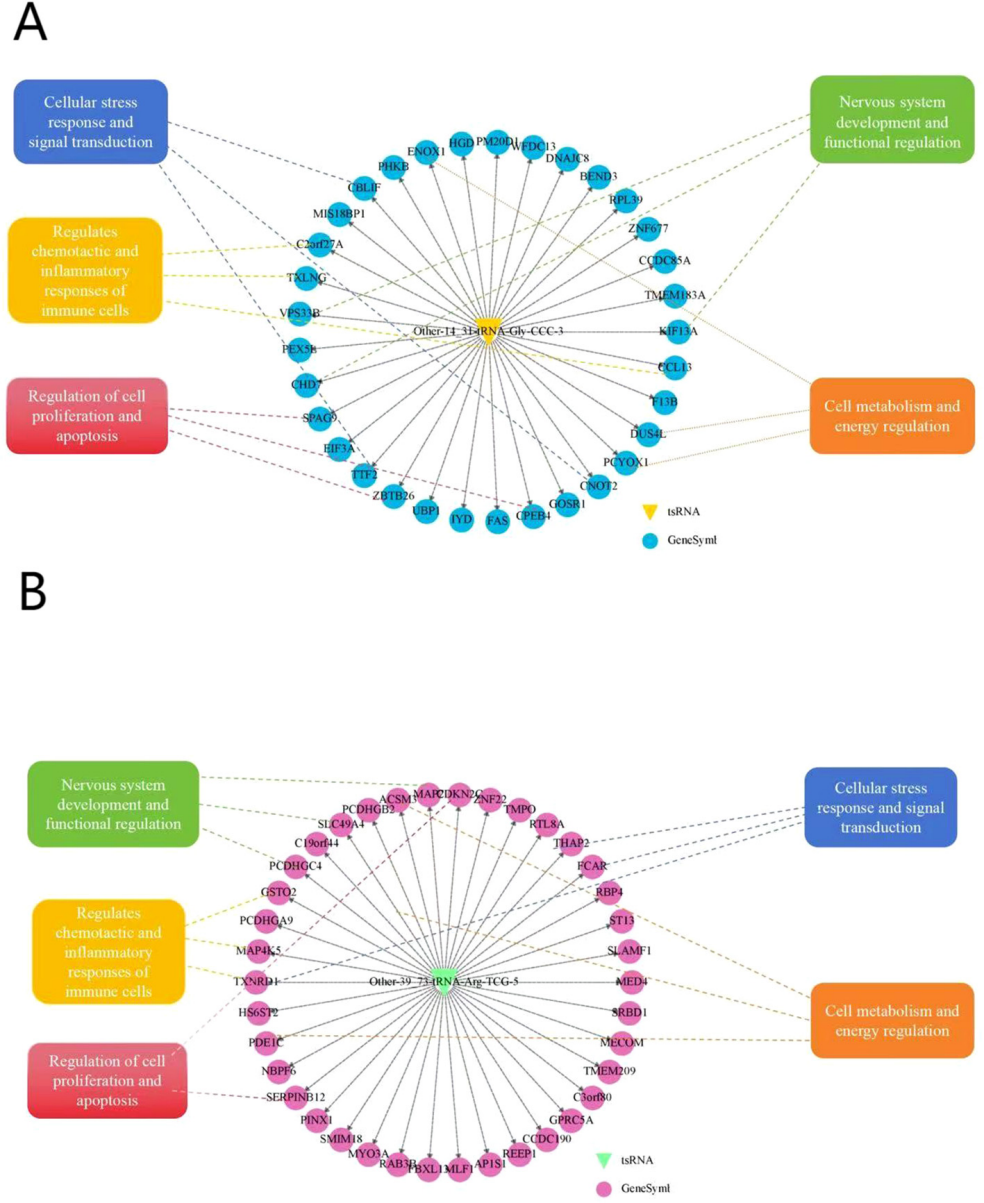
Targeted tsRNAs network construction.

In the tsRNA target gene prediction network shown in **Figure 11**, complex regulatory relationships between Other-14:31-tRNA-Gly-CCC-3 and Other-39:73-tRNA-Arg-TCG-5 and multiple potentialtarget genes can be observed (**Figures 11A, B**). These predicted target genes are radially distributed aroud tsRNA, suggesting that Other-14:31-tRNA-Gly-CCC-3 and Other-39:73-tRNA-Arg-TCG-5 may play a broad regulatory role in multiple biological processes. This figure presents a comprehensive molecular interaction network of various biological processes related to POD. The main aspects of this network include: cellular stress response and signal transduction pathways, represented by molecules such as FKAR, THAP2, MECOM, REEP1, which mediate the cellular response to surgical stress and conduct related signal cascades. Regulation of immune cell function, where molecules like CAR, GSTO2, SERPINB12, CCL13 are involved in regulating the chemotaxis and inflammatory response of immune cells. Regulation of cell proliferation and apoptosis, with factors such as CDKN2C, SERPINB12, MAP4K5 controlling the cell cycle process and programmed cell death, which is crucial for maintaining neuronal homeostasis. Regulation of nervous system development and function, where genes like PCDHGA9, MAP2, RAB3B are associated with neural development, synaptic function, and neural network regulation. The cross-regulation of these pathways may play an important role in the complex pathophysiological process of POD.

The entire network diagram is centered around a circle, and this center represents the target tsRNA. Each node represents a specific gene in the diagram. All kinds of genes and their related traits are regulated and interacted around this center.

## 5 Discussion

In our study, we performed tsRNA microarray detection on serum of clinical patients and found that tsRNA expression in POD patients was significantly different from that in Non-POD patients. Among the 90 differentially expressed tsRNAs, 34 were up-regulated and 56 were down-regulated. Two tsRNAs demonstrated the most striking expression differences between groups: Other-14: 31-tRNA-Gly-CCC-3 and Other-39: 73-tRNA-Arg-TCG-5. GO and KEGG pathway analyses of the target genes regulated by these differentially expressed tsRNAs identified key biological functions and signaling pathways involved in POD. Subsequent validation through RT-qPCR analysis of plasma samples in independent cohorts has provided initial evidence supporting the usefulness of these two tsRNAs as predictive biomarkers for identifying individuals at risk of developing POD. It is worth noting that this study used the PSM method to match the two groups of patients, achieving balance in age, ASA score, surgical duration, surgical type, and anesthesia method, and still found that the expression of Other- 14: 31-tRNA-Gly-CCC-3 and Other- 39: 73-tRNA-Arg-TCG-5 before surgery was positively correlated with the incidence of POD, and both showed extremely strong predictive ability in the ROC analysis. This comprehensive analysis provides novel insights into the molecular mechanisms underlying POD pathogenesis and highlights potential roles of specific tsRNAs in this condition.

Although the present investigation did not reveal statistically significant gender differences regarding the onset of POD, antecedent research has indicated that such gender differences may influence the onset of delirium (34). Consequently, this study stratified male and female subjects to mitigate the potential confounding effects associated with gender discrepancies. The AUC for Other-14:31-tRNA-GLy-CCC-3 and Other-39:73-tRNA-Arg-TCG-5 in the male cohort were recorded as 0.968 and 0.881, respectively. In the female cohort, the AUC values for Other-14:31-tRNA-Gly-CCC-3 and Other-39:73-tRNA-Arg-TCG-5 were determined to be 0.852 and 0.828, respectively. The findings indicated that both target tRNA species exhibited substantial predictive efficacy within both the male and female cohorts (**Supplementary Data Sheet 1**).

Furthermore, this study conducted a comprehensive analysis of preoperative clinical indicators relevant to the risk of postoperative delirium, including advanced age, ASA grade III, low BMI, and various laboratory parameters. Notably, lower hemoglobin levels, elevated CRP levels, reduced lymphocyte counts, decreased albumin concentrations, and increased D-dimer levels were found to correlate with a heightened risk of POD. Among these factors, albumin and lymphocyte count are frequently utilized in clinical practice as indicators of nutritional status (35–37). Our findings showed that POD patients had higher levels of CRP before surgery, which is consistent with reports that elevated markers of peripheral inflammation are associated with increased POD risk (38).Thus, these risk factors may reflect potential complications and an overall poor health status in patients that could elevate the likelihood of acute brain dysfunction during surgical procedures. These findings imply that abnormal regulation of tsRNA prior to surgery combined with the physiological risk states present in patients collectively heightens the susceptibility to acute brain dysfunction among elderly individuals undergoing surgery—thereby establishing an etiological basis for POD.

Bioinformatic enrichment analyses offered profound insights into the key pathways and processes that might modify postoperative delirium, indicating several potential pathophysiological mechanisms. The outcomes of the GO analysis disclosed that the biological processes associated with POD predominantly encompass numerous aspects such as intercellular adhesion communication, inflammatory response, and molecular signal transduction. In the analysis of biological processes, intercellular adhesion and other communication processes involve the connection between homologous and heterologous cells, which is intimately related to the reconstruction and repair of nerve cell networks in the brain (39). Our study found that the predicted target genes of Other-39:73-tRNA-Arg-TCG-5 include genes encoding microtubule-associated protein 2(MAP2). MAP2 is mainly located in the dendrites and cell bodies of neurons and interacts with microtubules through unique structural properties to stabilize the microtubule network and regulate its dynamics, which is crucial for neuronal morphogenesis, synaptic formation and neurotransmitter transmission. In addition,MAP2 is involved in the regulation of neuronal plasticity, which is the basis of higher cognitive functions such as learning and memory (40). Existing studies have indicated that MAP2 can inhibit tau protein deposition (41), and it is notable that the preoperative plasma tau protein concentration plays a significant role in POD recognition (42).

Synapse serves as a crucial communication mechanism between neurons and exemplifies a cell-cell adhesion process. Neuronal connectivity via synapses is essential for optimal brain function. Upon arrival of a nerve signal at the presynaptic membrane, neurotransmitters are released into the synaptic cleft, where they interact with postsynaptic receptors, thereby facilitating nerve signal transmission (43). Stressors, such as surgical interventions, may disrupt this process, potentially resulting in neuronal communication impairment and subsequent brain dysfunction. GO analysis of target tsRNA cells revealed significant synaptic structural components, including vesicle membranes and distal axons. Additionally, target gene enrichment analysis emphasized protocadherin (Pcdh), which is integral to both synapse formation and the regulation of specific neuronal connections (44, 45).

According to the neuroinflammatory hypothesis, the peripheral immune response triggered by surgical trauma can activate central nervous system cells and inflammatory mediators, resulting in impaired neuron and synaptic functions, thereby causing postoperative delirium (2). Circulating inflammatory cells and factors disturb the homeostasis of glial cells, synaptic plasticity, and neuronal function, which are the key processes in the pathogenesis of POD (46, 47). Additionally, some tsRNAs might exert significant regulatory roles in systemic and neuroinflammation (48, 49). The target gene TREM2 identified in our study regulates phagocytosis, inhibits inflammation, and amplifies neuroinflammatory signals, and its dissolved form sTREM2 can be used as a biomarker of POD (50, 51). Another target gene, CCL13, is involved in regulating immune cell activity and systemic inflammation, and is associated with frontotemporal dementia (52).

Furthermore, our KEGG pathway analysis demonstrated the enrichment of various metabolic and signaling pathways in tsRNAs that were differentially expressed in POD. Among these pathways, the most prominently enriched one was found to be associated with the metabolism of linolenic acid and linoleic acid, purine metabolism and GnRH signaling. This analytical investigation offers valuable insights into the biological pathways and processes that potentially contribute to the occurrence of POD.

The brain relies on key metabolic pathways to function properly. Several enriched pathways involve lipid and amino acid metabolism. These supply “fuel” and building blocks for brain cells. Problems in metabolic pathways like alpha-linolenic/linoleic acid metabolism could disrupt cell membranes or energy production in neurons and glia (53). This impacts how brain cells communicate. Alpha-linolenic and linoleic acids are essential omega-3 and omega-6 fatty acids that play a crucial role in the structural and functional integrity of the brain. These fatty acids are incorporated into the cell membranes of neurons, exerting their influence on membrane fluidity, permeability, and lipid raft organization (54). Additionally, alpha-linolenic acid enhances antioxidant defenses within the brain, thereby protecting neurons against oxidative damage that is associated with cognitive decline (55, 56). Any disruptions in the metabolism of these fatty acids may have negative consequences on cell membrane function, neurotransmitter systems, synaptic communication, and neuroinflammatory responses in patients suffering from delirium. Therefore, maintaining sufficient intake of these fatty acids may be beneficial in supporting optimal brain structure and function, particularly in older adults who are experiencing surgical stress (57).

This study has certain limitations. The sample size was relatively small. A larger sample size would enhance the accuracy. The study was carried out at a single center, which might introduce bias. Larger studies across multiple sites are requisite to fully comprehend tsRNAs and delirium. Certain factors were compared, yet adjustments were not made. There might be other variables that were not taken into account. The study merely provided evidence of a correlation. In order to validate the function of tsRNA and disclose specific regulatory mechanisms, this study needs to be further refined through animal experiments. At present, our study has preliminarily established an animal model of POD, and RT-qPCR was conducted to verify the expression of tsRNA-14:31 and tsRNA-39:73 in the animal model (**Supplementary Data Sheet 2**), which provides the direction for further research on POD, and more efforts are needed in the future to further clarify its mechanism.

## 6 Conclusion

This study discovered two new tsRNAs biomarkers, Other-14: 31-tRNA-Gly-CCC-3 and Other-39: 73-tRNA-Arg-TCG-5, which can predict the occurrence of POD in elderly orthopedic surgery patients. Before the surgery, the levels of these tsRNAs were significantly higher in patients with delirium compared to those without delirium. The analysis of the tsRNAs’ function using bioinformatics revealed their involvement in important neuronal and metabolic pathways related to cognition and brain health. The validation through propensity score matching confirmed the tsRNAs’ predictive capability, even after considering potential confounding factors.

## Data Availability

Raw data have been deposited to National Center for Biotechnology Information (NCBI) under the BioProject number PRJNA1233749.
