# Supplementary material for: Circulating tsRNAs serve as potential biomarkers for predicting postoperative delirium in elderly patients receiving lower extremity orthopedic surgery
Source: Front Psychiatry. 2025 Mar 26;16:1522984. doi: 10.3389/fpsyt.2025.1522984 (PMC11980442; doi:10.3389/fpsyt.2025.1522984)
Supplement: Supplementary file 1 [file DataSheet1.docx]

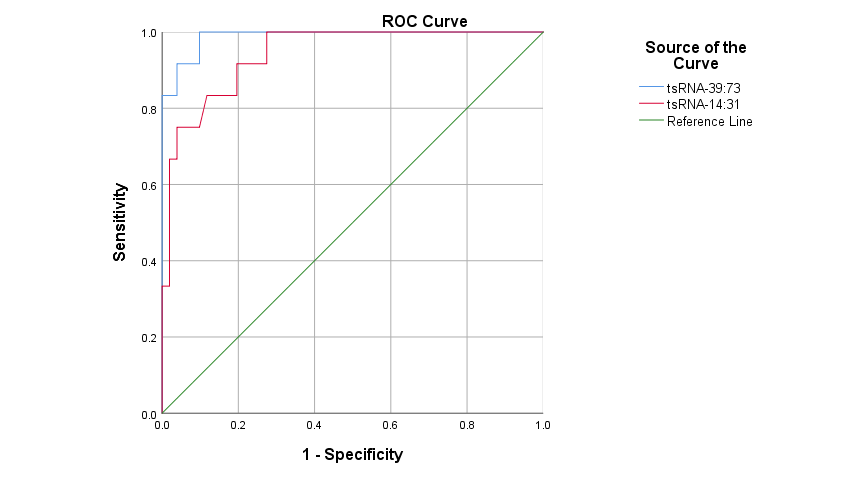


**Figure 1.** Diagnostic value of target tsRNA as a biomarker in male group


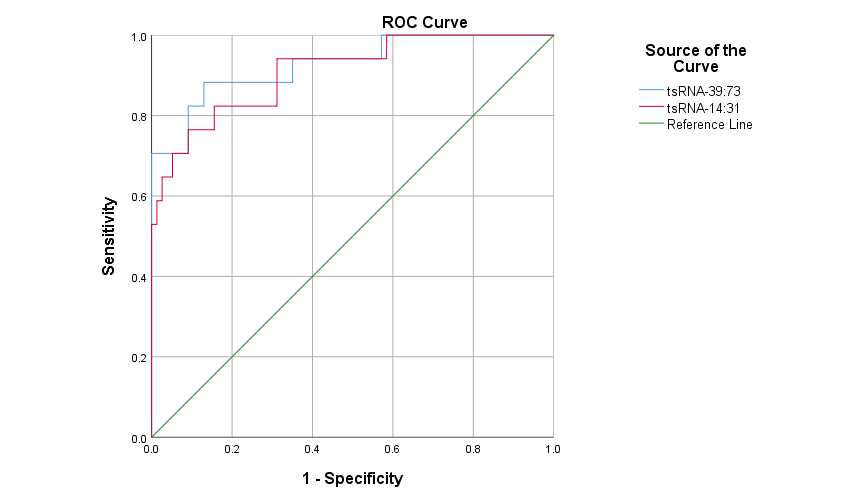


**Figure 2.** Diagnostic value of target tsRNA as a biomarker in female group

Although the present investigation did not reveal statistically significant gender differences regarding the onset of Postoperative Delirium (POD), antecedent research has indicated that such gender differences may influence the onset of delirium. Consequently, this study stratified male and female subjects to mitigate the potential confounding effects associated with gender discrepancies. The Area Under the Curve (AUC) for Other-14:31-tRNA-Gly-CCC-3 and Other-39:73-tRNA-Arg-TCG-5 in the male cohort were recorded as 0.968 and 0.881, respectively(Figure 1). In the female cohort, the AUC values for Other-14:31-tRNA-Gly-CCC-3 and Other-39:73-tRNA-Arg-TCG-5 were determined to be 0.852 and 0.828, respectively(Figure 2). The findings indicated that both target tRNA species exhibited substantial predictive efficacy within both the male and female cohorts.
