# Supplementary material for: Circulating tsRNAs serve as potential biomarkers for predicting postoperative delirium in elderly patients receiving lower extremity orthopedic surgery
Source: Front Psychiatry. 2025 Mar 26;16:1522984. doi: 10.3389/fpsyt.2025.1522984 (PMC11980442; doi:10.3389/fpsyt.2025.1522984)
Supplement: Supplementary file 2 [file DataSheet2.docx]

As an important functional area of memory, emotion, cognition, social interaction, and stress processing, the hippocampus plays an important role in neurocognition, and is associated with neurodegenerative and psychiatric diseases. Surgery and anesthesia can cause behavioral abnormalities, cognitive impairment, hippocampal inflammation, mitochondrial dysfunction, and neuron apoptosis in mice, suggesting that hippocampal function may be closely related to POD.

Therefore, in this study, a model of isoflurane anesthesia for lower abdominal surgery was established and the POD model was verified by behavioral means. RT-qPCR was performed on the hippocampal genes of POD group and CON group confirmed by behavioral means in mice, which laid a foundation for further exploring the mechanism of hippocampal participation in POD regulation.

**Experimental animals**: The animals utilized in the experiment were male mice of the C57 breed, aged 18 months. These mice were procured from Alingfei Biotechnology Co., LTD. It is noteworthy that all animal experiments conducted adhered to the Measures for the Management of Experimental Animals of Zhejiang Province. The Laboratory Animal Center of Ningbo University provided a Specific Pathogen Free (SPF) cell for the feeding of the mice. The temperature within the cell was effectively controlled at 22±2℃, while the humidity was maintained at 60±5%. Furthermore, the day and night cycle within the cell lasted for a duration of 12 hours. It is important to mention that the animals were housed in cages, with no more than 5 animals occupying each cage. The diligent staff ensured the timely provision of water and food particles. Notably, all animal behavior tests were conducted strictly in accordance with the specific room's experimental requirements. It is worth mentioning that all procedures followed the Guidelines for the Care and Use of Laboratory Animals as established by the USA National Institutes of Health (NIH). The Animal Ethics and Welfare Committee of Ningbo University (NBU20210003) duly approved these procedures.

**Methods of surgery/anesthesia and animal grouping:** The mice were allocated randomly into two groups: the delirium group (referred to as the POD group) and the control group (referred to as the CON group), with six mice in each group. In the POD group, the mice underwent an exploratory laparotomy procedure under the influence of isoflurane anesthesia. The mice were induced with isoflurane in an acrylic chamber for a duration of 15 minutes. Subsequently, the heads of the mice were placed in a maintenance mask to receive isoflurane and 100% oxygen. Following this, a simple exploratory laparotomy was performed by making a midline incision from the xiphoid process to 0.5cm above the symphysis pubis. This incision allowed for the exposure of the abdominal organs for a period of 2 minutes, after which the incision was sutured layer by layer. The entire operation lasted approximately 15 minutes. The mice were then returned to the anesthesia room and kept there for a duration of 2 hours. After recovering from the anesthesia, the mice were provided with water and food as per their normal routine. Lidocaine gel was applied to the wound every 8 hours until the conclusion of all experiments. Mice that exhibited abnormal behavioral changes following the surgery were categorized as POD mice. In contrast, the mice in the CON group were subjected to a similar environment as the POD group, spending 2 hours in an acrylic chamber during the anesthesia/surgery period, receiving 100% oxygen. However, no anesthesia or surgery was performed on the mice in the CON group.

**Buried food test (BFT)**:The primary parameter that was evaluated in this examination was the duration of incubation for the mice to ascertain concealed sustenance. The duration of incubation was designated as the interval between the moment the mice were introduced into the enclosure and the moment the nourishment particles were discovered. During the course of the experiment, the mice were deprived of sustenance for a span of 24 hours, albeit they were granted unrestricted access to water. On the following day, a 2-gram portion of daily sustenance was buried at one extremity of a pristine trial enclosure, situated 5 cm beneath the surface of the recently provided bedding. The placement of the animals and the concealment of the food pellets persisted unchanged. Commencing the experiment, the mice were positioned at the center of the enclosure and the duration taken by the mice to unveil the sustenance and clutch it using their anterior extremities/teeth was meticulously documented, that is, the incubation period of the mice's quest for nourishment.

**Open field experiment(OFT)**: The purpose of the experiment was to assess the mice's capacity for independent exploration, motor skills, and stress levels. The mice were placed within a square, open container with a black base measuring 50cm in length, 50cm in width, and 40cm in height. Positioned 2cm above the container, a camera captured the mouse's activity through wireless wifi-enabled photography. The experimenter then vacated the room. Subsequently, the recorded footage was analyzed using ANY-maze7.1 software to determine the distance covered by the mice and the duration spent within the central region.The central latency time reflects the anxiety of rats. The retention time in the central area of depressed rats also decreased significantly.

**Y Maze (New arm experiment )** :The Y maze is comprised of three cuboids, each with dimensions of 30cm×8cm×15cm (length × width × height). These cuboids are arranged at angles of 120° with respect to each other, and the background color is gray. The image acquisition system employed is the Yi Camera 1296p, while the image analysis system used is ANY-maze. The design of the Y maze is based on the characteristics that animals possess in their preference to explore new environments. To aid in spatial positioning, three black markers of different shapes (triangle, circle, and square) are affixed to the three arms of the maze. It should be noted that the positions of individuals and objects remain unchanged throughout the duration of the detection period.The new arm experiment was used to detect the space exploration ability of old mice.The experiment involved two distinct stages, with a time interval of 1 hour. The first stage, known as the training period, entailed separating the new arm with the use of a separator. Following this separation, the mice were placed in the starting arm and given the freedom to move between the starting arm and the other arms for a duration of 10 minutes. After an hour, the separator was removed. The second stage of the experiment involved placing the mice in the starting arm and allowing them to move freely between all three arms for a period of 5 minutes. Cameras were utilized to record both the time and the number of movements made by the mice in each arm

.
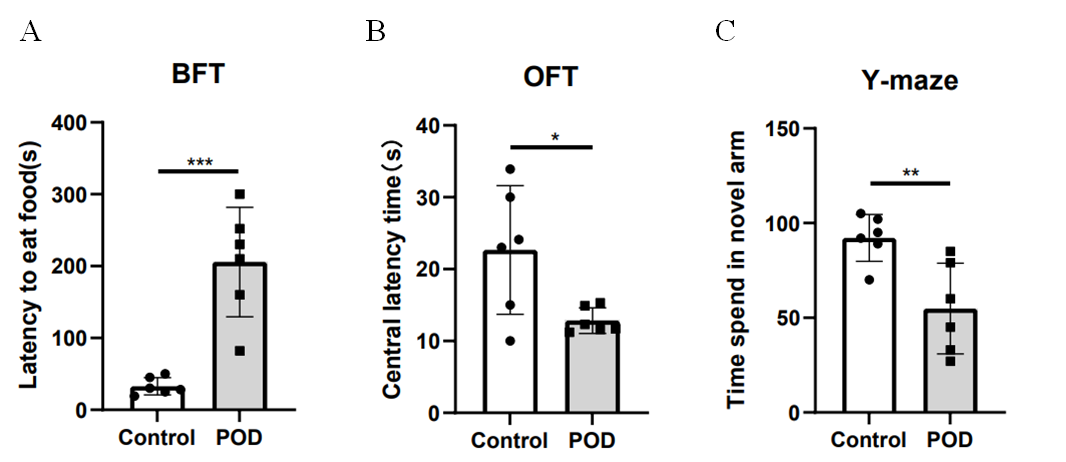


**Figure 1.**Behavioral experiment.(A)Buried food test (BFT):The duration of searching for hidden food in POD group was significantly longer than that in Control group;(B)Open field experiment(OFT):The central latency time of POD group was significantly lower than that of Control group;(C)Y Maze (New arm experiment ):The time spend in novel of POD group was significantly lower than that of Control group;* Significant at P≤0.05

**Result**

**Impaired learning and memory in delirious mice**

Food burial experiments have been used as one of the behavioral methods to detect POD. Compared with the Control group, the feeding latency of POD group mice was significantly prolonged, suggesting that the attention level of mice was changed after surgery(Figure1.A).

The central latency time of open field test used in this experiment is the inverse of the spatial cognitive ability of mice.The mice remained in the central cell longer if they had poor cognitive ability to perceive the new environment. In this experiment, the central latency time of POD group mice was significantly longer than that of Control group, indicating that the spatial cognitive ability of POD group mice declined(Figure1.B).Y Maze new arm exploration experiment to study spatial recognition memory ability, also using rodents like to explore new environments, tend to explore new novel arms preference. The longer and more times of exploring the novel arm, the stronger the spatial recognition and memory ability of mice.The exploration time of the new arm in POD group was significantly shorter than that in Control group(Figure1.C).

**Serum expressions of tsRNAs**


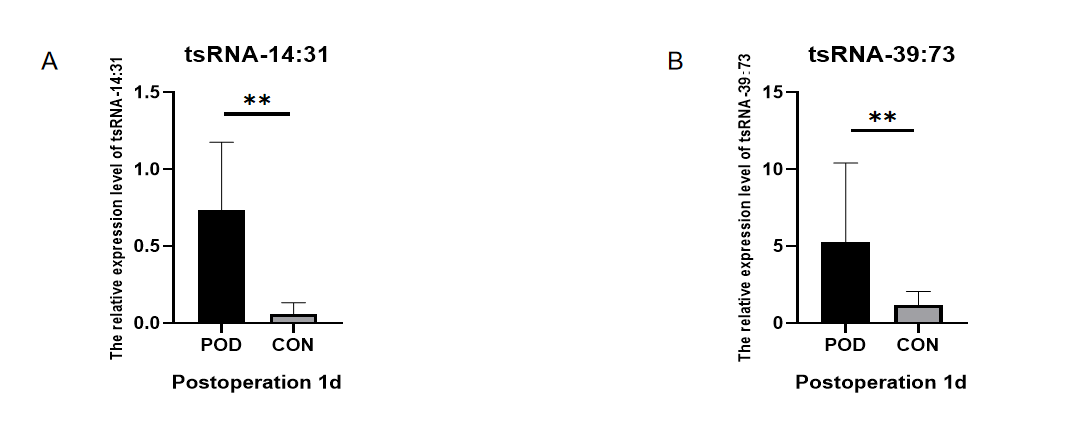


**Figure 2.** Relative expressions of circulating tsRNAs associated with POD in animal model. (A). The relative levels of tRF-14:31 of Control and POD groups from RT-qPCR. (B). The relative levels of tRF-39:73 of Control and POD groups from RT-qPCR. **Significant at P＜0.01

The levels of tRF-14:31-tRNA-Gly-CCC-3 and tRF-39:73-tRNA-Arg-TCG-5 in the hippocampus of mice in the POD and CON groups were examined through the utilization of RT-qPCR. As illustrated in Figures 2A and 2B, it was observed that mice with POD displayed higher levels of tRF-14:31 and tRF-39:73 as opposed to mice without POD (P<0.01). In conclusion, the augmentation of plasma levels of tRF-14:31-tRNA-Gly-CCC-3 and tRF-39:73-tRNA-Arg-TCG-5 following surgery were linked with the occurrence of POD.
